# Three-Dimensional Magnetic Resonance Cholangiopancreatography for the Diagnosis of Biliary Atresia in Infants and Neonates

**DOI:** 10.1371/journal.pone.0088268

**Published:** 2014-02-05

**Authors:** Bo Liu, Jinhua Cai, Ye Xu, Xuehua Peng, Helin Zheng, Kaiping Huang, Jing Yang

**Affiliations:** Department of Radiology, Children's Hospital of Chongqing Medical University, Chongqing, China; Vanderbilt University, United States of America

## Abstract

**Background and Objective:**

Magnetic resonance cholangiopancreatography (MRCP) is widely accepted for visualization of the biliary system. However, the sensitivity and specificity of MRCP for the diagnosis of biliary atresia (BA) are still not fully elucidated. This study aimed to investigate the diagnostic value of three-dimensional MRCP (3D-MRCP) for BA in a large cohort of cholestatic infants and neonates.

**Methods:**

One hundred ninety patients with infant jaundice underwent 3D-MRCP and one or more of the following: (1) intraoperative cholangiography, (2) laparoscopic exploration and pathological examination, or/and (3) clinical therapy. Statistical analyses were performed to determine the diagnostic accuracy of 3D-MRCP for BA.

**Results:**

Our study demonstrated that 158 of 190 patients were interpreted as having BA by 3D-MRCP; of those, 103 patients were confirmed as having BA, whereas 55 patients did not have BA. Of the 32 patients interpreted as non-BA cases by 3D-MRCP, one patient was misdiagnosed. The diagnostic accuracy for 3D-MRCP was 70.53% (134 of 190), the sensitivity was 99.04% (103 of 104), the specificity was 36.05% (31 of 86), the negative predictive value was 96.88% (31 of 32), the positive predictive value was 65.19% (103 of 158), the positive likelihood ratio was 2.7473, the negative likelihood ratio was 0.0267, and the Youden index was 0.3509.

**Conclusions:**

The sensitivity of 3D-MRCP in diagnosing BA was excellent, but the specificity was not as high as described in previous reports. 3D-MRCP can be an effective screening method but should be combined with other modalities to identify BA and distinguish it from other causes of infant jaundice.

## Introduction

Biliary atresia (BA), whose etiology remains unknown, is a condition unique to infancy that causes destructive, idiopathic, and inflammatory processes in both the intrahepatic and extrahepatic bile ducts and finally results in fibrosis, obliteration of the biliary tract, and biliary cirrhosis. It is the most frequent surgically correctable liver disorder in infancy and the most frequent indication for liver transplantation in pediatric patients [1]. If untreated, BA progresses to cirrhosis, with portal hypertension and liver failure leading to death within two to three years. There have been encouraging results in treating this disease since the Kasai operation, which can restore bile flow through a reconstructed hepatic portoenterostomy to a jejunal loop, was first used to treat BA in 1959, such that the operation has become the first-line treatment. The success of the surgery depends largely on the age at which it is performed. It is generally accepted that the Kasai operation is more successful in children when performed earlier than 60 days of age. However, early identification and timely surgery, which are crucial for better prognoses, remain challenging [2]–[5].

Magnetic resonance cholangiopancreatography (MRCP) is a widely accepted method of imaging the biliary system. Previous reports have documented the diagnostic value of MRCP for BA by reporting an accuracy of 82–98%, sensitivity of 90–100%, and specificity of 77–96% [6]–[9]. In these studies, infantile hepatitis (IH) was the typical disease that could be differentiated from BA. However, other conditions, such as biliary stenosis (BS) and breast milk jaundice (BMJ), can also influence biliary secretion and possibly cause nonvisualization of the extrahepatic biliary tree, thereby leading to false-positive BA diagnoses. Therefore, the diagnostic value, particularly the specificity of MRCP for BA, may have been overestimated in previous studies. To investigate the diagnostic value of MRCP for BA in infants and neonates, this study retrospectively analyzed the three-dimensional MRCP (3D-MRCP) findings of a large infant cohort with consecutive cholestasis and compared them with intraoperative cholangiography (IC), laparoscopic exploration and pathological examination (LEPE), or/and clinical outcomes.

## Materials and Methods

### Ethics Statement

The study protocol was approved by the Human Ethics Committee of the Children's Hospital of Chongqing Medical University. Written informed consent was obtained from the parents or guardians of all patients before the examinations.

### Patients

Between January 2008 and July 2010, 190 patients (111 males and 79 females; median age, 69 days; age range, from 20–330 days) with infant jaundice and clinical suspicion of BA underwent 3D-MRCP. Before the MRCP examination, each subject was sedated orally with chloral hydrate (50 mg/kg) or intramuscularly with phenobarbital (5 mg/kg). All 3D-MRCP studies were performed after a fast of at least 4 h. All patients subsequently underwent IC, LEPE, and/or clinical therapy. The clinical follow-up exceeded more than one year.

### Data collection

The studies were performed with a 1.5-T magnetic resonance imaging (MRI) unit (Signa Propeller HD; GE Medical Systems, Milwaukee, WI, USA) using a single-channel quadrature head coil or eight-channel phased-array cardiac body coil. The supine and feet-advanced positions were used for all patients. Images were obtained in both the axial and coronal planes.

The routine axial-plane T2- and T1-weighted images were first obtained. In all 3D-MRCP studies, the fast-recovery fast-spin echo-accelerated pulse sequence was used with the following parameters: the repetition time ranged from 2,000 to 6,000 ms, the echo time ranged from 96.6 to 2057.1 ms, the slice thickness ranged from 1.2 to 2.6 mm, the slice gap ranged from an overlap of −0.6 to −1.3 mm, the field of view ranged from 24 to 36 cm and covered the diaphragmatic dome and the C loop of the duodenum, and the array spatial sensitivity encoding technique was used to accelerate the imaging process. The original coronal images of 3D-MRCP were reconstructed with a maximum intensity projection algorithm using post-processing reformatting software on a workstation (ADW4.3; GE Medical Systems). The extrahepatic central biliary ducts (including the right, left and common hepatic ducts), common bile duct, and gallbladder were best shown through the upper procedures.

### Statistical Methods and Data Analysis

Statistical analyses were performed using the Statistical Product and Service Solutions version 13 (SPSS Inc., Chicago, IL, USA) software. The McNemar test was used to compare the coincidence of the findings of 3D-MRCP and the IC, LEPE, and clinical outcomes. *P*<0.05 was considered statistically significant.

The following criteria were defined to interpret the 3D-MRCP findings. A non-BA diagnosis was assigned if the extrahepatic central biliary ducts and common bile duct were visualized. BA was diagnosed if any portion of the extrahepatic central biliary duct or common bile duct could not be delineated, regardless of whether the gallbladder was presented. The 3D-MRCP images were interpreted in consensus by two experienced pediatric radiologists, who were blinded to the clinical information.

## Results

### MRI manifestations

**Figure 1:**
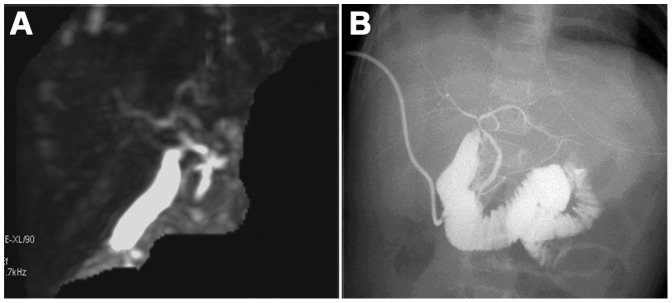
Infantile hepatitis in a 52-day-old boy. The extrahepatic central biliary ducts (including the right, left, and common hepatic ducts) and common bile duct are visualized on 3D-MRCP (A), which is consistent with the intraoperative cholangiography (B).

In 32 cases, the MRCP findings were interpreted as non-BA according to the visualization of the extrahepatic central biliary ducts (right, left, and common hepatic ducts) and common bile duct (Figure 1A). The gallbladder was visible on the 3D-MRCP images in all cases.

**Figure 2:**
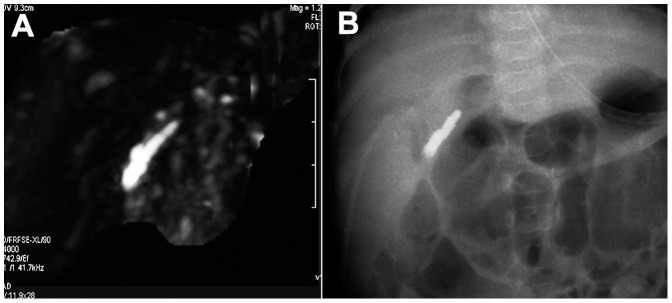
Biliary atresia in a 76-day-old female infant. 3D-MRCP does not display the extrahepatic central biliary ducts and common bile duct (A). Intraoperative cholangiography confirms a diagnosis type III BA (B).

**Figure 3:**
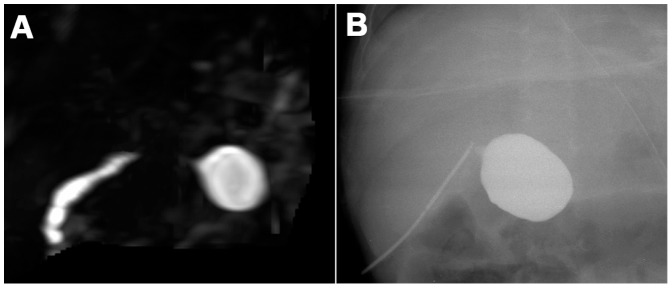
Biliary atresia in a 90-day-old female infant. 3D-MRCP does not display the extrahepatic biliary ducts except for the cystic common bile duct (A). The infant undergoes intraoperative cholangiography and is confirmed as having type III BA (B).

In the remaining 158 cases, in which the extrahepatic central biliary ducts and common bile duct partially or completely disappeared on the 3D-MRCP images, the MRCP findings were interpreted as BA (Figures 2A and 3A). In 13 cases, in which the common hepatic duct was not visible, whereas the common bile duct, cystic duct, and gallbladder were visible or disappeared, the MRCP findings were interpreted as type II BA. In the other 145 cases, in which the entire extrahepatic central biliary ducts (right, left, and common hepatic ducts) and common bile duct were not visible, the MRCP findings were interpreted as type III BA. Of the 158 cases, the gallbladder was visible on the 3D-MRCP images in 106 cases and not visible in 52 cases.

### The findings of intraoperative cholangiography (IC), laparoscopic exploration and pathological examination (LEPE), and clinical therapy

Eighty-six patients were diagnosed as non-BA by IC or clinical therapy, of which 44 patients were diagnosed as having BS, nine patients had IH (Figure 1B), 23 patients had cytomegalovirus hepatitis (CMVH), six patients had BMJ, two patients had pathologic jaundice (PJ), and two patients had cholestasis. In these patients, the jaundice was cured or alleviated after clinical therapy. Six patients were diagnosed by IC as having type I BA, in which the distal bile duct was atretic and the common hepatic duct, gallbladder, and cystic duct were normal. Twenty-four patients were diagnosed by IC as having type II BA, in which the common hepatic duct was atretic at different levels, whereas the common bile duct, cystic duct, and gallbladder were patent or atretic. Seventy-four patients were diagnosed by IC or LEPE as having type III BA, in which the entire extrahepatic biliary system, including the common hepatic duct, gallbladder, and common bile duct, was atretic (Figures 2B and 3B). Type III BA was the most common type and occurred in 71.2% of the BA cases (Table 1).

**Table 1:** Findings of IC, LEPE and CCT for the 190 patients.

	BA			Non-BA					
	Type I	Type II	Type III	BS	IH	CMVH	BMJ	PJ	Cholestasis
IC	6*	24	10*	44	4	3			1*
		(1▴+23*)		(10▴+34*)	(1▴+3*)	(1▴+2*)			
LEPE			64*						
CCT					5	20	6	2▴	1▴
					(3▴+2*)	(10▴+10*)	(3▴+3*)		
Count	104			86					
	(1▴+103*)			(31▴+55*)					

Note: ▴ represents how many cases were diagnosed for non-BA with 3D-MRCP.

*represents how many cases were diagnosed for BA with 3D-MRCP. Abbreviations: IC, intraoperative cholangiography; CCT, cure after clinical therapy; LEPE, laparoscopic exploration and pathological examination; BA, biliary atresia; BS, biliary stenosis; IH, infantile hepatitis; CMVH, cytomegalovirus hepatitis; BMJ, breast milk jaundice; PJ, pathologic jaundice.

### Comparison of the 3D-MRCP findings with those of Intraoperative Cholangiography (IC), Laparoscopic Exploration and Pathological Examination (LEPE), and clinical therapy

Of the 32 patients whose MRCP findings were interpreted as non-BA, 31 patients were confirmed as non-BA by IC or clinical therapy, and only one patient was confirmed as having BA by IC. Of the 158 patients whose MRCP findings were interpreted as having BA, 103 patients were confirmed as having BA by IC or LEPE and 55 patients were confirmed as having non-BA (Table 2).

**Table 2:** Comparison of the 3D-MRCP findings and the final diagnoses for 190 infants and neonates.

	Diagnosis of 3D-MRCP for non-BA (n = 32)		Diagnosis of 3D-MRCP for BA (n = 158)	
Final diagnoses	No. of findings	Method of confirmation	No. of findings	Method of confirmation
BA	1	IC	103	IC (39) and LEPE (64)
BS	10	IC	34	IC
IH	4	IC (1) and CCT (3)	5	IC (3) and CCT (2)
CMVH	11	IC (1) and CCT (10)	12	IC (2) and CCT (10)
BMJ	3	CCT	3	CCT
Cholestasis	1	CCT	1	IC
PJ	2	CCT		

Note: The data in the parentheses represent the number of cases confirmed by the indicated method. Abbreviations: IC, intraoperative cholangiography; CCT, cure after clinical therapy; LEPE, laparoscopic exploration and pathological examination; BA, biliary atresia; BS, biliary stenosis; IH, infantile hepatitis; CMVH, cytomegalovirus hepatitis; BMJ, breast milk jaundice; PJ, pathologic jaundice.

IC, LEPE, and clinical outcomes were used as the diagnostic gold standard for BA. The sensitivity, specificity, positive predictive value (PPV), negative predictive value (NPV), positive likelihood ratio (PLR), negative likelihood ratio (NLR), and Youden index of 3D-MRCP for the diagnosis of BA are summarized in Table 3.

**Table 3:** Comparison of the 3D-MRCP findings and the gold-standard findings.

		3D-MRCP		
		BA	Non-BA	count
gold standard	BA	103	1	104
	Non-BA	55	31	86
	count	158	32	190
P Value	<0.001			
R Value	0.467			
Kappa Value	0.371			
sensitivity	99.04%	(103 of 104)		
specificity	36.05%	(31 of 86)		
accuracy	70.53%	(134 of 190)		
NPV	96.88%	(31 of 32)		
PPV	65.19%	(103 of 158)		
PLR	2.7473	(99.04%/36.05%)		
NLR	0.0267	[(1–99.04%)/36.05%]		
Youden index	0.3509	99.04%+36.05%−1		

Note: Intraoperative cholangiography, cure after clinical therapy, and laparoscopic exploration and pathological examination were used as the gold standards for diagnosis of BA. Abbreviations: BA, biliary atresia; PPV, positive predictive value; NPV, negative predictive value; PLR, positive likelihood ratio; NLR, negative likelihood ratio.

## Discussion

BA is a serious disease that endangers the lives of infants and neonates, and its differentiation from IH remains a common diagnostic problem because IH results in similar clinical presentations, such as jaundice, pale stools, and hepatomegaly [1], [5], [10]. However, the therapeutic methods for BA and IH are completely different: the former is treated with surgical therapy, including the Kasai operation or liver transplantation, as early as possible, whereas the latter is treated with medical therapy. Early diagnosis and younger age at time of surgery afford the patients with BA a higher likelihood of a relatively good outcome. This study demonstrated that 3D-MRCP, which permitted non-invasive and precise evaluation of the biliary tree, could be used as an alternative imaging modality in the diagnosis of BA in infants and neonates.

Many reports have suggested that MRCP is a well-established non-invasive modality for visualizing the biliary system, including the first branch of the intrahepatic biliary ducts, extrahepatic bile ducts, and gallbladder [10]. In our study, however, the first branch of the intrahepatic bile ducts was not visible on 3D-MRCP images. This lack of visibility may have resulted from insufficient bile and/or the diameter of the biliary duct in neonates and infants being too small. Thus, we used the visibility of the extrahepatic biliary ducts on 3D-MRCP images as the diagnostic criterion for non-BA. In contrast, we considered the absence of any portion of the extrahepatic biliary ducts as the diagnostic criterion for BA. With the advances in MRI techniques, such as the application of parallel imaging technology, we can obtain MRCP images with higher spatial resolution, shorter scanning time, and less motion blurring [10]. In our study, the fast-recovery fast-spin echo-accelerated pulse sequence used in 3D-MRCP imaging was built on the foundation of the 3D fast-spin echo pulse sequence. This pulse sequence has been developed primarily to enable high-resolution images for MRCP studies. The fast recovery feature is designed to enhance the intensity of fluid that has long T2-relaxation time and shorten the repetition time. The shortened repetition time makes the high-resolution images practical. The average imaging time of 3D-MRCP was 174 s for the respiratory-triggered cases and 180 s for the non-respiratory-triggered cases. Furthermore, 3D-MRCP scans within a slab that covers the intrahepatic and extrahepatic biliary ducts. The 3D nature of the scan implies that the slice-selecting gradient is cancelled, and only one frequency-encoding gradient and two phase-encoding gradients are used to create overlapping thin slices of the target area with a sufficiently small slice thickness and gap. Therefore, 3D-MRCP is suitable for the evaluation of jaundice in neonates and infants.

The sensitivity and specificity of MRCP in the diagnosis of BA differed from those reported in previous studies. An earlier study recruited 26 cases and reported that MRCP had an accuracy of 82%, sensitivity of 90%, specificity of 77%, PPV of 75%, and NPV of 91% in the identification of BA by using the same strict criteria, which required delineation of the right, left, common hepatic, and common bile ducts [9]. In contrast with previous reports, we found that 3D-MRCP had higher sensitivity and NPV in the identification of BA, but the specificity and PPV were lower. A false negative diagnosis of BA was made in only one case. In this case, the right, left, and intrahepatic biliary ducts were observed, but the common hepatic and common bile ducts were not observed by using IC. Through retrospective analyses, we determined that we incorrectly identified the high signals of the intestinal tract as the common hepatic and common bile ducts. In contrast, a false positive diagnosis of BA was made in 55 patients. In 45 patients who were diagnosed as having type III BA by 3D-MRCP, the extrahepatic central biliary ducts and common bile duct were not observed. In eight patients who were diagnosed as having type II BA by 3D-MRCP, only the common bile duct was observed. In two patients who were diagnosed as having type II BA by 3D-MRCP, the right, left, and common bile ducts were observed, except for the common hepatic biliary duct. These 55 cases indicate a potential pitfall in the diagnosis of BA with 3D-MRCP. Visualization of the biliary system relies on sufficient production and excretion of bile. In this study, most of the false positive cases were cases of BS. In such a condition, the extremely small diameter of the hypoplastic bile duct and inadequate fluid typically make the duct invisible on MRCP images [9], [11], [12]. Visualization of a very thin bile duct might be possible with the application of a higher-magnetic-field MR scanner and further improvement in the resolution of MRCP.

In this study, we found that the type of BA diagnosed by 3D-MRCP was not reliable. Twenty-one patients who were diagnosed as having type III BA by 3D-MRCP were confirmed as having type II BA by IC. Six patients who were diagnosed as having type III BA by 3D-MRCP were confirmed as having type I BA by IC. There appeared to be no correlation between the types of BA indicated by the MRCP and IC findings. Additionally, the lack of visibility of the gallbladders interpreted by the 3D-MRCP images was also not consistent with the IC findings. The gallbladder was not visible on the 3D-MRCP images in 52 cases, of which the gallbladder was confirmed as small by IC or US in 51 cases and absent in only one case. Although a small gallbladder on MRCP images can be used as a suggestive sign of BA [12], we did not measure the gallbladder size on MRCP images because the abdominal US can measure the gallbladder size more accurately. Further research should explore the relationship between the size of the gallbladder and BA.

A multidisciplinary approach for the diagnosis of BA and differentiation from other causes of jaundice has been suggested [13]–[15]. In our hospital, the following steps are typically taken to evaluate jaundice in infants and neonates: first, direct and indirect reacting serum bilirubin measurement; then, non-invasive imaging modalities, including ultrasonography (US), scintigraphy, and/or MRCP; and finally, invasive methods, such as liver biopsies. A finding of hyperbilirubinemia typically suggests a diagnosis of BMJ, for which further examination is not necessary. In this study, however, six cases of BMJ were included in the MRCP studies because the aim of the study was to investigate the diagnostic accuracy of MRCP in a group of consecutive patients with jaundice. In these six cases, three cases were interpreted as BA on MRCP images, which might partially contribute to the lower specificity in this study. For non-invasive imaging modalities, US is convenient and widely available and is typically recommended as the initial imaging procedure. Imaging via abdominal US can help discern structural abnormalities, such as choledochal cysts. A small or absent gallbladder on hepatic US suggests BA, but the relatively low sensitivity of US indicates that it cannot be used to exclude this diagnosis. Recent studies have concluded that the triangular cord sign, which is identified at the porta hepatis and likely represents the fibrosis at the portal plate, was 73–100% sensitive and 98–100% specific for the diagnosis of BA [16]–[18]. If no such characteristic US sign is visualized, hepatobiliary scintigraphy or MRCP is the next step. Hepatobiliary scintigraphy can reliably exclude the diagnosis of BA by indicating the drainage of radiotracer into the small bowel. The sensitivity of scintigraphy for the diagnosis of BA was demonstrated to be 83–100%, whereas the specificity was relatively lower, ranging from 70–80% [19], [20]. In rare cases, bile drainage is initially present in infants with cholestatic disease but is subsequently lost after two to three weeks of age. MRCP should be used in such cases. There are some common benefits offered by MRCP and US, including shorter examination time, no pretreatment, and no ionizing radiation. Compared with MRCP, the US is lower cost and typically does not require any oral or intramuscular sedation. Hepatobiliary scintigraphy is also less expensive than MRCP, and there is no need for sedation for the diagnosis of BA in infants and neonates. Therefore, US, hepatobiliary scintigraphy, and MRCP have advantage in the evaluation of infant jaundice. However, abdominal US and hepatobiliary scintigraphy should still be included in the first-line imaging examination of infants with cholestatic jaundice given the cost and need for sedation. When patients are not suitable for US or hepatobiliary scintigraphy examination, 3D-MRCP may be used as an alternative modality.

Duodenal drainage is another method that has been used to help ensure correct diagnosis of BA [16], [17], [19], [21]–[25]. However, the procedure of duodenal drainage is cumbersome, and strict criteria for the test have not been defined. Some authors have suggested that stool color cards for BA screening are reliable and easy. The sensitivity of the screening is relatively low, whereas the specificity is high [26]–[29]. Our study demonstrated that the sensitivity of 3D-MRCP in the identification of BA was considerably higher than that of the stool color card screening, although the specificity was lower. Combining 3D-MRCP with stool-color-card screening could increase the diagnostic sensitivity and specificity for BA.

Liver biopsy is invasive but helpful in the differentiation between BA and IH and has a reported sensitivity of up to 100% and a specificity of 75.9% for BA diagnosis [30], [31]. In our hospital, liver biopsy is reserved for infants with high suspicion of BA but negative findings from non-invasive imaging methods. In this study, liver biopsy was performed in only five cases. All cases were diagnosed as BA, which was coincident with the results of IC. Endoscopic retrograde cholangiopancreatography (ERCP) is another invasive modality for the evaluation of jaundice in infants. According to previous reports, ERCP has a high sensitivity and specificity in BA diagnosis but is subject to substantial morbidity (0.8–7.0%), technical failure (3–9%), and occasional mortality (0.05–1%) [32], [33]. With the advances in non-invasive imaging modalities, ERCP has not been considered a choice of diagnostic methods for infant jaundice due to its invasive nature.

To obtain rapid and high-quality images, sedation is required for the patients who underwent MRCP. In our study, the patients were sedated with orally administered chloral hydrate or intramuscularly injected phenobarbital. No side effects were detected in our patients. According to previous reports, however, the adverse effects of these sedative drugs included respiratory depression, airway obstruction, agitation, ataxia, vomiting, and cardiac arrhythmia [34]–[36]. Recently, Maitre et al. [37] reported that increased exposure to phenobarbital is associated with serious neurodevelopmental outcomes. Furthermore, chloral hydrate and phenobarbital also have limitations, such as unpredictable onset, long duration, and the lack of a reversal agent, which make them less-than-ideal sedatives. In future MRCP examinations, general anesthesia should be preferred for small children because its safety and success are predictable.

In conclusion, the specificity of 3D-MRCP for BA is not as high as described in previous reports. If any portion of the biliary tree is not visualized, the 3D-MRCP findings must be interpreted in conjunction with other diagnostic and clinical information (such as US, hepatobiliary scintigraphy, and stool color cards) to distinguish BA from other causes of infant jaundice. However, 3D-MRCP can still be considered an effective screening technology for BA in infants and neonates before surgery, particularly when patients are not suitable for US or hepatobiliary scintigraphy examination.
